# Case Report: Diagnostic challenges in pediatric appendicitis: a case of perforated appendicitis with secondary hepatic abscess

**DOI:** 10.3389/fped.2026.1784237

**Published:** 2026-02-20

**Authors:** Xueke Li, Li Lin, Cheng Yu, Li Zhang, Yilian Duan

**Affiliations:** 1 Department of Ultrasound Medicine, Union Hospital, Tongji Medical College, Huazhong University of Science and Technology, Wuhan, China; 2 Clinical Research Center for Medical Imaging in Hubei Province, Wuhan, China; 3 Hubei Province Key Laboratory of Molecular Imaging, Wuhan, China

**Keywords:** abdominal pain, acute appendicitis, case report, hepatic abscess, pediatric

## Abstract

Appendicitis in young children (age <5 years) frequently presents with atypical symptoms, creating diagnostic challenges in primary care and emergency settings. This report describes a 4-year-old girl who presented to the emergency department with fever, vomiting, and abdominal pain. Initial evaluation including abdominal ultrasound showed no evidence of appendicitis, and she was discharged with a presumptive diagnosis of viral gastroenteritis. Five days later, she was readmitted with persistent symptoms and markedly elevated inflammatory markers. Advanced imaging revealed a hepatic abscess, initially attributed to primary liver pathology. Diagnostic laparoscopy ultimately revealed perforated appendicitis with secondary hepatic abscess. The patient underwent successful laparoscopic appendectomy and abscess drainage with complete recovery. This case highlights the essential clinical approach of maintaining a broad differential diagnosis, recognizing the limitations of imaging, and mandating systematic re-evaluation with structured follow-up when a patient's expected clinical improvement fails to materialize.

## Introduction

Acute appendicitis remains one of the most common pediatric surgical emergencies, yet its diagnosis in young children continues to present significant challenges. Children under 5 years of age often present with atypical symptoms including poorly localized abdominal pain, vomiting, and fever that can mimic common viral illnesses (1). This atypical presentation contributes to perforation rates of 30%–50% in this age group, substantially higher than rates observed in older children and adults. Perforated appendicitis can result in serious complications including diffuse peritonitis, intra-abdominal abscesses, and septic portal vein thrombosis (2). Secondary hepatic abscess represents a particularly rare but potentially life-threatening complication of appendiceal perforation, occurring in fewer than 0.1% of appendicitis cases (3). The rarity of this complication, combined with its dramatic clinical presentation, can mislead clinicians away from the underlying appendiceal pathology.

Diagnostic imaging plays a central role in evaluating suspected appendicitis, yet each modality has inherent limitations. Ultrasound (US) remains the preferred initial imaging study due to absence of ionizing radiation, but its diagnostic accuracy varies considerably based on operator experience, patient body habitus, and disease stage (4). Advanced imaging with computed tomography (CT) or magnetic resonance imaging (MRI) offers improved diagnostic accuracy but raises concerns about radiation exposure, cost, and availability in emergency settings (5).

This case report describes a young child who presented with nonspecific symptoms initially managed as viral gastroenteritis, subsequently found to have perforated appendicitis complicated by hepatic abscess. The case illustrates the diagnostic challenges inherent in evaluating young children with abdominal complaints and emphasizes the importance of systematic re-evaluation when the clinical course deviates from expected trajectories.

## Case description

A previously healthy 4-year-old girl presented to the emergency department with a one-day history of fever (maximum temperature 39.2 °C), recurrent non-bilious vomiting, and diffuse abdominal pain. The child was able to localize discomfort to the epigastric region but could not provide more specific characterization. There were no reported diarrhea, urinary symptoms, or respiratory complaints. Her parents denied recent travel, sick contacts, or unusual dietary exposures. Physical examination revealed an uncomfortable but alert child with mild diffuse abdominal tenderness on palpation, most pronounced in the epigastrium and right upper quadrant. There was no guarding, rebound tenderness, or palpable masses. Bowel sounds were present and normal. The remainder of the examination, including cardiovascular and respiratory systems, was unremarkable.

Given the constellation of fever, vomiting, and abdominal pain, the emergency department clinicians conducted laboratory studies and imaging. Initial laboratory evaluation showed leukocytosis (white blood cell count 12.5 × 10^9^/L). Abdominal ultrasound was performed which showed the appendix was visualized and appeared normal with no evidence of inflammation, wall thickening, or periappendiceal fluid. Abdominal radiographs showed no obstruction or free air. Based on the clinical presentation and physical examination, a working diagnosis of viral gastroenteritis was established. The patient received intravenous hydration and antiemetic therapy in the emergency department with symptomatic improvement, and was discharged home.

Five days after the initial emergency department visit, the patient returned due to persistent fever and progressive abdominal pain. The child had no significant past medical history. There were no previous surgeries, trauma, or blood transfusions. Both parents were alive and healthy with no family history of genetic disorders, inflammatory bowel disease, or recurrent infections. On admission, the patient appeared ill with persistent fever (38.8 °C). Abdominal examination now revealed tenderness in the right upper quadrant but no rebound tenderness. Laboratory investigations demonstrated marked leukocytosis (30.41 × 10⁹/L), severe elevation of C-reactive protein (185.27 mg/L), thrombocytosis (511 × 10⁹/L), neutrophilia (88.20%), lymphopenia (9.7%), and absolute neutrophil count of 26.82 × 10⁹/L (Table 1).

**Table 1:** Laboratory findings on admission.

Laboratory Test	Result	Reference Range	Interpretation
White Blood Cell Count	30.41 × 10⁹/L	4.4–11.9 × 10⁹/L	Elevated
Neutrophil Percentage	88.20%	22%–65%	Elevated
Absolute Neutrophil Count	26.82 × 10⁹/L	1.2–7.0 × 10⁹/L	Markedly Elevated
Lymphocyte Percentage	9.70%	23%–69%	Decreased
Platelet Count	511 × 10⁹/L	188–472 × 10⁹/L	Elevated
C-Reactive Protein (CRP)	185.27 mg/L	<4 mg/L	Markedly Elevated

**Figure 1:**
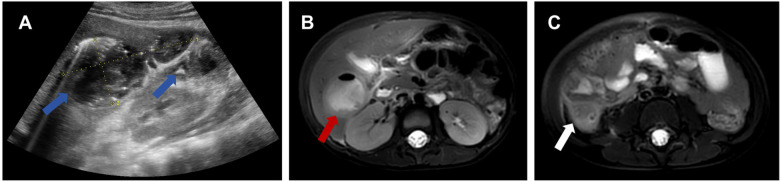
**(A)** Abdominal ultrasound demonstrates the heterogeneous gas-containing lesion in the right hepatic lobe (blue arrows), indicative of abscess formation. **(B)** Axial MRI obtained using an early tumor differentiation enhancement protocol shows a low T1, high T2 signal mass in the posterior right hepatic lobe (red arrow). **(C)** Axial MRI reveals a mass shadow in the appendix region (white arrow), consistent with inflamed appendix.

Abdominal ultrasound revealed a complex cystic-solid lesion in the liver consisting of two adjacent heterogeneous masses measuring approximately 3.3 × 2.4 cm and 2.1 × 0.9 cm in the subcapsular region of the posterior inferior right hepatic lobe. Retroperitoneal ultrasound identified retroperitoneal lymphadenopathy. Chest CT showed minimal abnormal density in the lower lobes of both lungs, suggestive of reactive changes. MRI of the liver with contrast enhancement revealed a mass in the posterior right hepatic lobe (the larger lesion measured approximately 41 × 34 × 40 mm) with imaging characteristics suggestive of hepatic abscess (Figure 1). MRI did not clearly visualize the appendix but revealed a mass adjacent to its expected region (Figure 1C). Based on the imaging findings, clinical presentation, and markedly elevated inflammatory markers, the working diagnosis at this time was primary hepatic abscess with systemic inflammatory response. Given the diagnostic uncertainty, persistent systemic inflammation despite broad-spectrum antibiotics, and clinical deterioration, the decision was made to proceed with diagnostic laparoscopy.

Laparoscopic exploration revealed extensive intra-abdominal pathology. The key findings included: acute perforated appendicitis with gangrenous changes, periappendiceal abscess formation with extensive fibrinous adhesions, diffuse suppurative peritonitis with purulent fluid throughout the peritoneal cavity, and a thin-walled abscess on the visceral surface of the right hepatic lobe in direct communication with the subhepatic space. The surgical team performed laparoscopic adhesiolysis to free the inflamed appendix from surrounding structures, appendectomy, drainage of the periappendiceal abscess, incision and drainage of the hepatic surface abscess with copious irrigation, and placement of subdiaphragmatic drains. Intraoperative findings confirmed that the hepatic abscess was secondary to the perforated appendix rather than a primary liver pathology. The immediate postoperative course was complicated by persistent fever on postoperative day one. By postoperative day three, the patient became afebrile with significant reduction in white blood cell count to 20.05 × 10⁹/L and resolution of abdominal pain. She received intravenous broad-spectrum antibiotics (piperacillin-tazobactam) and supportive care including fluid replacement and nutritional support. Histopathological examination of the appendectomy specimen confirmed acute phlegmonous appendicitis with transmural inflammation and periappendicitis. On postoperative day six, follow-up ultrasound showed the drainage tube in appropriate position with surrounding hypoechoic areas consistent with resolving inflammation in the liver. The patient was discharged on postoperative day seven with oral antibiotics to complete a 14-day course.

One month after discharge, the patient was seen in the outpatient clinic with a one-day history of recurrent vomiting. The differential diagnosis at this time included post-operative ileus (though timing was atypical), residual intra-abdominal infection, medication-related gastritis from prolonged antibiotic therapy, or unrelated viral gastroenteritis. Physical examination showed a well-appearing child with mild abdominal tenderness but no peritoneal signs. CT scan of the abdomen was performed, which revealed nodular and speckled hyperdense shadows at the junction of the ascending colon and inferior segment of the right hepatic lobe with ill-defined margins from the hepatic parenchyma, consistent with postoperative changes and resolving inflammation, and complete resolution of the previously noted abscess in the inferior segment of the right hepatic lobe. The patient was admitted for 24 h of observation, received wound care, and was started on oral cephalosporin for presumed gastritis. At three-month follow-up, the patient remained well with complete resolution of all symptoms and normal physical examination.

## Discussion

This case illustrates several important principles in the diagnosis and management of appendicitis in young children, particularly regarding diagnostic reasoning in the face of uncertainty, recognition of imaging limitations, and the importance of systematic re-evaluation when clinical improvement fails to occur. Appendicitis in children under 5 years of age presents diagnostic challenges distinct from those encountered in older children and adults. Young children often cannot articulate the classic symptom progression of periumbilical pain migrating to the right lower quadrant. Instead, they may present with nonspecific symptoms including fever, vomiting, and poorly localized abdominal pain that can easily be attributed to more common diagnoses such as viral gastroenteritis (6, 7). In this case, the initial presentation of fever, vomiting, and diffuse abdominal tenderness with negative ultrasound reasonably suggested viral illness rather than surgical pathology—reasonable given that viral conditions predominate in pediatric emergencies. The challenge for clinicians lies not in achieving perfect diagnostic accuracy on initial presentation, but rather in recognizing when the clinical course deviates from expected patterns and warrants reassessment. Our patient's persistent fever beyond 72 h and progressive rather than improving symptoms triggered appropriate concern for alternative diagnoses. For negative imaging with lingering suspicion, earlier follow-up may have identified deterioration sooner.

Ultrasound remains the preferred initial imaging modality for suspected pediatric appendicitis due to its lack of ionizing radiation, real-time imaging capabilities, and widespread availability (8). However, ultrasound has significant limitations that can lead to false-negative results. The sensitivity of ultrasound for pediatric appendicitis exceeds 89%, with substantial variation based on operator expertise, patient body habitus, bowel gas interference, and anatomic factors such as retrocecal or subhepatic appendiceal position (7, 9, 10). In early appendicitis before significant inflammation develops, the appendix may appear normal or may not be adequately visualized. Technical factors including patient cooperation, degree of abdominal guarding, and presence of overlying bowel gas further complicate sonographic evaluation in young children. Critically, a negative ultrasound does not exclude appendicitis, particularly when clinical suspicion remains elevated (11). In this case, the initial ultrasound visualized a normal-appearing appendix, which provided reassurance but did not definitively rule out early appendicitis. As the disease progressed to perforation over the subsequent days, the clinical picture became more complex with secondary manifestations overshadowing the primary appendiceal pathology.

When ultrasound is inconclusive and clinical suspicion for appendicitis persists, advanced imaging with CT or MRI should be considered. The choice between these modalities involves weighing diagnostic accuracy, radiation exposure, availability, and practical considerations (12). CT scanning provides excellent visualization of the appendix and periappendiceal region with specificity exceeding 81% for appendicitis diagnosis (13). Modern low-dose CT protocols have substantially reduced radiation exposure while maintaining diagnostic accuracy, addressing longstanding concerns about cumulative radiation risk in children (5). The decision to use CT should consider the patient's age (younger children are more radiation-sensitive), body habitus (CT is particularly valuable in obese patients where ultrasound is limited), and the urgency of diagnosis (CT is faster and more readily available than MRI in most emergency settings). From a cost perspective, CT represents a middle ground between ultrasound and MRI, though institutional pricing varies considerably (7, 14, 15). CT is particularly valuable in identifying complications of appendicitis including perforation, abscess formation, and alternative diagnoses. MRI is particularly useful when ultrasound is nondiagnostic but clinical suspicion remains high, and in situations where radiation exposure is particularly concerning (16). However, MRI has longer acquisition times, higher costs, requires greater patient cooperation (which may necessitate sedation in young children), and may have limited availability in emergency settings, particularly during nights and weekends. In resource-limited settings or community hospitals, the availability of MRI and radiologist expertise in pediatric interpretation may be significant limiting factors. Some institutions may lack 24 h MRI access or pediatric-trained radiologists, necessitating transfer to tertiary centers for advanced imaging. These logistical considerations must be weighed against the clinical urgency and stability of the patient (17). MRI identified the hepatic abscess and showed a periappendiceal mass, though the appendix was not clearly visualized. This highlights an important principle: even advanced imaging may not reveal the complete clinical picture when presentations are atypical or when secondary complications dominate the radiologic findings. The dramatic presentation of a large hepatic abscess drew attention away from the smaller, perforated appendix that was the primary source of infection.

Hepatic abscess secondary to appendiceal perforation is exceedingly rare in children, occurring in fewer than 0.1% of appendicitis cases (3). In contemporary practice, improved diagnostic techniques and earlier surgical intervention have further reduced this already uncommon complication. However, when it does occur, the dramatic presentation of the hepatic abscess can overshadow the underlying appendiceal pathology, leading to misdiagnosis as primary liver infection. Several mechanisms can explain the development of hepatic abscess following appendiceal perforation. Direct contiguous spread can occur when an inflamed, perforated retrocecal or subhepatic appendix directly contacts the liver capsule, as likely occurred in our patient (18). Alternatively, organisms can reach the liver via the portal venous system in cases of pylephlebitis (septic thrombophlebitis of the portal vein), a pathway documented in several pediatric case reports (19, 20). Less commonly, biliary tract spread can occur. The rarity of this complication means that clinicians may not consider appendicitis in the differential diagnosis of pediatric hepatic abscess, particularly when typical signs of appendicitis are absent and initial imaging focuses attention on the liver pathology. Wichmann and colleagues reported that four of five patients with appendicitis-related hepatic abscess lacked typical appendiceal symptoms, and the appendiceal pathology was not identified on initial imaging (21). This pattern of “hidden appendicitis” masked by dramatic secondary manifestations emphasizes the importance of comprehensive surgical exploration when diagnostic uncertainty persists.

The decision to proceed with diagnostic laparoscopy rather than limiting treatment to abscess drainage reflected appropriate consideration of alternative etiologies. The decision to proceed with diagnostic laparoscopy proved crucial and aligns with current evidence-based guidelines for managing diagnostically uncertain cases with persistent inflammation and clinical deterioration (12). Diagnostic laparoscopy serves a dual function: it provides definitive visualization of intra-abdominal pathology when imaging is equivocal or misleading, and it allows immediate therapeutic intervention when surgical pathology is identified (22). In this patient, laparoscopy revealed the complete picture of perforated appendicitis with secondary hepatic surface abscess that had not been fully appreciated on preoperative imaging. This allowed for comprehensive treatment including appendectomy, abscess drainage, and peritoneal lavage in a single procedure. The alternative approach of treating the hepatic abscess in isolation (with percutaneous drainage or antibiotics alone) would have failed to address the underlying appendiceal source, likely resulting in treatment failure and further complications.

The most important lesson from this case is not that the initial evaluation was inadequate, but when clinical improvement does not occur as expected, clinicians must maintain an open differential diagnosis, avoid premature closure on initial impressions, and consider alternative or evolving pathology. This approach balances the need to avoid excessive testing in self-limited illnesses with the imperative to identify surgical emergencies in a timely manner.

## Study limitations

This case report has several limitations that should be acknowledged. As a single case report from a tertiary pediatric surgical center, the findings have inherent limitations in generalizability and cannot support conclusions about prevalence or optimal management strategies for similar cases. The follow-up period extends only three months, as the patient was lost to subsequent contact despite attempts to obtain long-term outcome data. This relatively brief observation period prevents assessment of delayed complications such as adhesive bowel obstruction, incisional hernia formation, or recurrent intra-abdominal infection that may manifest months or years following complex abdominal surgery. The retrospective nature of the analysis introduces potential documentation bias, and the initial imaging studies were performed at an outside facility, limiting our ability to review technical factors that may have contributed to the false-negative ultrasound examination. Additionally, the diagnostic approach employed in this case—including ready access to MRI, pediatric-trained radiologists, experienced pediatric surgeons, and resources for diagnostic laparoscopy—may not be feasible in community hospitals or resource-limited settings, which affects the reproducibility of our management strategy. Finally, the precise timeline of disease progression from early appendicitis to perforation remains unclear, as we cannot determine whether this occurred rapidly within 24–48 h or evolved gradually over the five-day interval between presentations.

## Conclusions

This case of perforated appendicitis with secondary hepatic abscess in a 4-year-old child demonstrates that systematic re-evaluation is essential when clinical improvement fails to materialize. The successful diagnosis and management of this rare presentation underscores three critical principles: maintaining high clinical suspicion despite initially negative imaging, recognizing that ultrasound cannot definitively exclude appendicitis in young children, and utilizing diagnostic laparoscopy when imaging findings remain discordant with clinical deterioration. Through adherence to these principles, clinicians can effectively navigate diagnostic uncertainty and achieve favorable outcomes even in complex pediatric cases.

## Data Availability

The original contributions presented in the study are included in the article, further inquiries can be directed to the corresponding authors.
